# Analysis approaches for the identification and prediction of *N*^6^-methyladenosine sites

**DOI:** 10.1080/15592294.2022.2158284

**Published:** 2022-12-23

**Authors:** Yuwei Yang, Zhiyu Liu, Junru Lu, Yuqing Sun, Yue Fu, Min Pan, Xueying Xie, Qinyu Ge

**Affiliations:** a State Key Laboratory of Bioelectronics, School of Biological Science and Medical Engineering, Southeast University, Nanjing, People’s Republic of China; b Department of Pathology and Pathophysiology School of Medicine, Southeast University, Nanjing, China

**Keywords:** m6A methylation, analytical tools, detection methods, prediction

## Abstract

The global dynamics in a variety of biological processes can be revealed by mapping transcriptional m^6^A sites, in particular full-transcriptome m^6^A. And individual m^6^A sites have contributed to biological function, which can be evaluated by stoichiometric information obtained from the single nucleotide resolution. Currently, the identification of m^6^A sites is mainly carried out by experiment and prediction methods, based on high-throughput sequencing and machine learning model respectively. This review summarizes the recent topics and progress made in bioinformatics methods of deciphering the m^6^A methylation, including the experimental detection of m^6^A methylation sites, techniques of data analysis, the way of predicting m^6^A methylation sites, m^6^A methylation databases, and detection of m^6^A modification in circRNA. At the end, the essay makes a brief discussion for the development perspective in this area.

## Introduction

The term ‘epigenetics’ was coined by Waddington in 1942. It was used to define the study to discover the mechanism of the correlation between phenotypes and genotypes and the relationship of this mechanism to the mechanics of development revealed by experimental embryology [1]. However, Waddington was unable to explain the molecular processes associated with genes, as it took two years for Avery to identify DNA as genetic material [2]. Over the last 80 years, with revolutionary findings of molecular mechanisms of epigenetic control [3], nowadays epigenetic is recognized as the study of hereditary changes without altering the underlying DNA sequence, it focuses on how behaviours and the environment can bring about changes that affect the functioning of genes.

As an important field of research in epigenetics, relative to DNA and histone modification, understanding of RNA modification is still in its early stages. Although the pseudouridine (Ψ), the most abundant modification found in total RNA from human cells, was discovered only 2 years after the first modified DNA nucleoside. It was not until 2012 [4] that the domain of deciphering RNA modifications got its own name, ‘Epitranscriptome.’ More than 170 different types of chemical RNA modifications have already been found on coding and non-coding RNAs (ncRNAs) to date, most of which are methylation modifications [5]. The focus on the effect of RNA modifications in the aspect of gene regulation has already been as important as the modification of DNA and histone.

*N^6^*-methyladenosine (m^6^A), the methylation of the sixth carbon atom of adenosine, was firstly found in polyadenylated RNA from mammalian cells in the 1970s [6]. Currently m^6^A has been considered the most abundant and best studied post-transcriptional modification in mammalian messenger and non-coding RNA. Because mRNA contains genetic information, previous studies regarding m^6^A modification mostly focused on mRNA. However, recently the crosstalk between circRNAs and m^6^A is beginning to gain attention. The RNA modification process for m^6^A methylation is catalysed by a multi-component methyltransferase complex named ‘m^6^A writers.’ There are four methyltransferases generating m^6^A in separate RNAs respectively in the mammalian transcriptome [7]. The ribosomal m^6^A RNA modification is mediated by the *N^6^*-Methyladenosine methyltransferase ZCCHC4 [8] and the METTL5-TRMT112 complex [9]. And the m^6^A in the U6 small nuclear RNA (snRNA) are catalysed by METTL16 [10]. It is remarkable that the m^6^A in the MAT2A mRNA sequences which encodes the enzyme responsible for S-adenosylmethionine (SAM) biosynthesis are also formed by METTL16. Finally, a large portion of m^6^A deposition on mRNA and other transcriptions derived from RNA polymerase II is formed by the heterodimer complex METTL3-METTL14 [11,12]. The primary function of METTL3 is the catalytic core, while METTL14 provides a platform for RNA-binding [13]. RNA modifications were considered to be static and immutable following their covalent attachment in the past. In 2012, the confirmation of physiological substrate and function of fat mass and obesity-associated (FTO) protein [14] provide a breakthrough insight into the state of RNA modification. Meanwhile, as the best physiological substrate for FTO, m^6^A in nuclear RNA was deemed to the first dynamic and reversible RNA modification [15], making the argument more compelling. Demethylases included FTO and ALKBH5 are also called ‘eraser’ because of their catalysing function of removing m^6^A from the RNA sequence. m^6^A have a profound effect on the maturation process of mRNA closely related to gene expression, including pre-mRNA treatment, nuclear exportation of mRNA [16], mRNA stability [17], and translation efficiency [18,19]. The dominant mechanism by which m^6^A carries out its biological function m^6^A exerted is through the recruitment of specific proteins. These specific proteins are called ‘reader protein.’ The m^6^A reader proteins have two distinct mechanisms for recognizing m^6^A: direct reading and indirect reading. Cytoplasmic proteins the family of domain YTH, YTHDF1, YTHDF2, YTHDF3, bind directly m^6^A with their eponymous YTH domains [17,20]. Indirect reading is caused by the decreasing of the energy between m^6^A:U base pairs and A:U base pairs, this energy difference reduces the structural stability of RNA, which may change the binding between RNA and protein [21]. Two types of heterogeneous nuclear ribonucleoprotein, HNRNPC [22] and HNRNPG [23], are indirect m^6^A reader proteins. In addition to recruiting reader proteins for transcription, m^6^A on chromosome-associated regulatory RNA (carRNAs) modulate gene expression by regulating the transcription of neighbouring mRNA [24]. The extensive effects of m^6^A on gene expression imply essential roles on numerous physiological and pathophysiological. The fundamental function of m^6^A in brain development [25], skeleton function [26] and immune system development [27] has been revealed by analysis of tissue-specific knockout mouse models of METTL3 and METTL14. Furthermore, the knockout of either METTL3 or METTL14 has a serous influence on differentiation in various stem cell or progenitor cell systems [28,29]. Currently, it has been confirmed that the pathogenesis of a variety of diseases is associated with m^6^A [30,31].

Dynamic m^6^A regulators have demonstrated the high research potential of m^6^A methylation. There are tens and thousands of m^6^A sites across the transcriptome. Methods that map m^6^A transcription sites, especially whole-transcriptome m^6^A sites, could uncover the potential global dynamic changes during different biological processes. The stoichiometric information obtained on single nucleotide resolution could be used to assess the contributions of individual m^6^A sites to biological functions. The METTL3/METTL14 methytransferase have the remarkable characteristic that the specificity of depositing m^6^A on the transcriptome. Previous studies have determined that the consensus motif is commonly occurring DRACH (D = G/A/U, R = G/A, H = A/U/C) consensus sequence [32]. However, the percentage of methylated DRACH motifs is only 5%, which may have an impact on site recognition. In addition, the distribution of m^6^A in the transcriptome showed an obvious regional deviation. m^6^A can be found throughout the transcription length, but is strongly enriched near the stop codon and in abnormally long internal exons [33,34]. According the property and context sequence of m6A, many approaches have been developed to reveal the map of the m6A modification. Biophysical and biochemical methods have been primary means of identifying and quantifying the m6A modification nucleotide by nucleotide in the past. As a large number of high-throughput datasets have been accumulated, bioinformatics has been a cost-effective approach to deciphering epitranscriptome. The review systematically summarized the emerging topics and recent progress in bioinformatics methods to decipher the m^6^A methylation, including experimental m^6^A methylation sites detection, related data analysis techniques, m^6^A methylation sites prediction methods, identification of m^6^A sites from circular RNA and m^6^A methylation databases.

## Experimental methods for *N*^6^-methyladenosine sites identification

**Figure 1.**
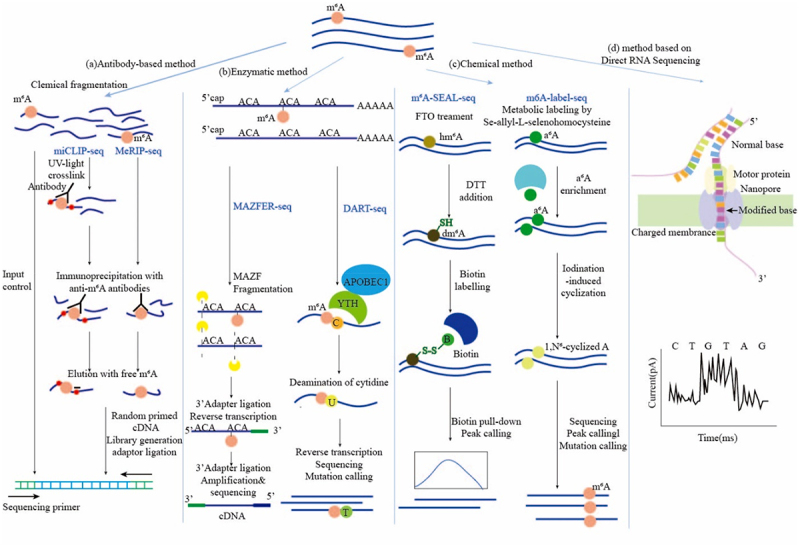
Experimental strategies in detection of m^6^A methylation sites.

The increasing size of the data and much larger RNAs have made it difficult and inefficient to detect the sequence context of modifications, advancement of the field has been stagnant for a long time. The application of high-throughput techniques that based on the detection and quantification of RNA modification has brought about a breakthrough (Figure 1). Current experimental methods are based on next-generation sequencing, but reverse transcription may introduce errors. Nevertheless, this problem could be solved by single-molecule direct sequencing, with the result that this technology is beginning to attract attention in the detection of m^6^A sites (Table 1).

**Table 1.** Evaluation of experimental methods for m^6^A sites identification and quantification.

Methods		Resolution	Advantages	Limitations
Antibody-based Method to Identify *N*^6^-Methyladenosine Site	MeRIP-seq(m^6^A-seq)^[33,35]^	100 ~ 200nt	·Low amount of input material required;	·Inability to distinguish m^6^A from m^6^A_m_ ·Low reproducibility
	miCLIP-seq^[36]^	Single- nucleotide	·Detection of multiple m^6^A sites within the same peak. ·Ability to distinguish m^6^A from m^6^A_m._ ·Without pretreatment of cells with modified nucleotides	·High quantity of mRNA material required ·Complex library protocol ·Low crosslinking yield
	PA-m^6^A-seq^[37]^	Single- nucleotide	·Compatible with investigation on nucleic acid–protein interactions.	·Low crosslinking yield
	m^6^A-LAIC-seq^[39]^	100 ~ 200·nt	·Elucidate tissue or cell-state specificity of m^6^A levels	·Empirical titration of the antibody concentration required
Enzymatic Methods to Identify *N*^6^-Methyladenosine Sites	MAZTER-seq^[41]^	Single-nucleotide	·Provides the m^6^A stoichiometry information ·Low false- positive rate	·Only detects m^6^A sites in an ACA sequence context ·Inability to distinguish ACA sites in close proximity
	m^6^A-REF-seq^[42]^	Single-nucleotide	·Direct identification method	·Only detects m^6^A sites in an ACA sequence context
	DART-seq^[44]^	Single-nucleotide	·Limited input RNA material ·Versatility to the approach	·Low sensitivity for low-abundance m^6^A sites
	m^6^A-SAC-seq^[46]^	Single-nucleotide	·A quantitative method ·Limited input RNA	·A motif preference of GAC over AAC.
	m^6^ACE-seq^[47]^	Single-nucleotide	··Precise methylomes uniquely mediated by each methyltransferase/demethylase ·mapping m^6^A_m_	·motif specificity
Chemical Methods to Identify *N*^6^-Methyladenosine Sites	m^6^A-label-seq^[50]^	Single-nucleotide	·High accuracy ·Direct identification method	·Low labelling yield and labelling time window scale ·Lack stoichiometric information
	m^6^A-SEAL^[53]^	100–200nt	·Can be optimized for detection of cap m6Am ·High sensitivity and specificity ·Direct identification method	·Lack stoichiometric information
Methods based on Direct RNA Sequencing to Identify *N*^6^-Methyladenosine Sites	Nanopore	Single-nucleotide	·No PCR biases ·Direct measurement of the number of m6A sites per isoform	·High general base error rate ·Method never applied to cellular mRNAs ·High cost
	SMRT	Single-nucleotide	·Direct investigation of m6A relation to isoform and transcript features ·Direct measurement of the number of m6A sites per isoform	·High base error rate ·Low sensitivity level ·High cost

### Antibody-based method to identify N^6^-methyladenosine site

The most common methods to detect *N*^6^-Methyladenosine site at the transcriptome-wide scale depend on a combination of immunoprecipitation and next-generation sequencing. The Methyl-RNA-immunoprecipitation-sequencing (MeRIP-seq) [35] and m^6^A-seq [33] are two of the most representative technologies. Although MeRIP-seq and m^6^A-seq were developed by two independent research groups, initial RNA preparation and the IP step are very similar. Poly(A)+-selected RNA was fragmented into 100 ~ 200-nucleotide-long oligonucleotides, parts of these RNA fragments were immunoprecipitated by anti-m^6^A affinity purified antibody, others were input control. Afterwards, immunoprecipitated as well as input control fragments were prepared as libraries, and subjected to massively parallel sequencing.

Nevertheless, only 100 ~ 200 nucleotides-length m^6^A modified regions could be detected by MeRIP-seq. Not being able to identify the precise location of m^6^A in mRNA may limit the understanding of the potential for m^6^A modification in the regulation of gene expression. Consequently, the specific mutational signatures induced by crosslinking antibodies to m^6^A modification before reverse transcription have been taken advantage of developing new methods. Most crosslinks of antibodies are induced by ultraviolet light (UV). m^6^A individual-nucleotide-resolution crosslinking and immunoprecipitation sequencing (miCLIP-seq) [36] mappings m^6^A in human and mouse mRNA at single-nucleotide resolution by the UV-induced mutational signatures of m^6^A on the opposite sites in complementary DNA (cDNA) during reverse transcription. Analogously, photo-crosslink-assisted m^6^A-sequencing (PA-m^6^A-seq) [37] was developed from photoactivatable ribonucleoside-enhanced crosslinking and immunoprecipitation (PAR-CLIP) [38], which make use of the photoreactivation of 4-thiouridine (4SU) or 6-thioguansine (6SG).After incorporated into mRNA, 4SU could covalently crosslink with residues of nearby aromatic amino acid in RNA-binding proteins upon 365 nm UV irradiation, leading the transition of T-to-C in PCR step.

Quantitative information of m^6^A represents the proportion of m^6^A-modified transcripts of each gene in the total transcription, which is very important for the assessment of m^6^A regulatory impact. m^6^A-level and isoform-characterization sequencing(m^6^A-LAIC-seq) [39] does not fragment the RNA prior to anti-m^6^A RNA immunoprecipitation (RIP) to keep full-length RNAs. Besides, m^6^A-LAIC-seq links the differences in alternative polyadenylation (APA) usage to m^6^A methylated transcriptions, revealing diverse proportions of m^6^A-modified transcriptions between the mRNA isoform.

To date, antibody-based methods have already made the identification and quantification of m^6^A across transcriptome range possible. In consideration of cost and efficiency, despite MeRIP-seq holds disadvantages of low reproducibility [40], high false positives and limited quantitative information, it is still adopted by most research institutes compared to other methods.

### Enzymatic methods to identify N^6^-methyladenosine sites

The cleavage specificity of RNases could be used to map m^6^A modifications in mRNA. The ability of an Escherichia coli toxin and RNA endoribonuclease, MazF, has been utilized by MAZFER-seq [41] and m^6^A-sensitive RNA-endoribonuclease-facilitated sequencing (m^6^A-REF-seq) [42] to specifically cleave the unmethylated ACA motif at 5’termina, leaving methylated (m^6^A)CA motifs intact [43]. These methods have the characteristic of precision and could allow to transcriptome-wide identify m^6^A sites in single-base resolution. Moreover, the two methods have their own characteristics. The m^6^A stoichiometry information at ACA motifs on mRNAs was provided by MAZFER-seq. And the conservation of m^6^A sites in mammals was revealed by m^6^A-REF-seq applied to detect distribution patterns of m^6^A modifications in different tissues of human, mouse and rat. Enzymatic methods largely reduce the requirement of starting RNA amount. Therefore, they could be applied to identify m6A sites for rare samples from pathological tissues or early embryos. However, because MazF is specific to the ACA motif at 5’termina, only 16–25% of all methylated sites could be identified.

The mutational signatures of m^6^A during reverse transcription could be generated by the reaction between specific enzymes and m^6^A or its reader proteins. In deamination adjacent to RNA modification target sequencing (DART-seq) [44], mutations at sites adjacent to m^6^A sites were induced by the conjugate of cytidine deaminase APOBEC1 and m^6^A reader protein YTHDF. Nevertheless, the narrow spatial distance between m^6^A and the adjacent sites possibly leads to bias m^6^A detections. Likewise, reverse transcriptase during reverse transcription could be induced by *N*^1^, *N*^6^-cyclized-m^6^A [45]. m^6^A-selective allyl chemical labelling and sequencing(m^6^A-SAC-seq) [46] converts m^6^A into *N*^6^-allyl, *N*^6^-methyladenosine(a^6^m^6^A) by Dim1/KsgA family of dimethyltransferases. After iodination-induced cyclization reaction, a^6^m^6^A is transferred to *N*^1^, *N*^6^-cyclized-m^6^A. However, m^6^A-SAC-seq shows a motif preference of GAC over AAC, which might result in the loss of m^6^A sites detection. Furthermore, the combination of enzymatic and antibody-based methods also enables to identify m^6^A modification at a single-base resolution.m^6^A-Crosslinking-Exonuclease-sequencing(m^6^ACE-seq) [47] generate a comprehensive atlas of disparate methylomes uniquely mediated by every individual known methyltransferase or demethylase. And m^6^ACE-seq highlights the importance of m^6^A_m_ in the detection of m^6^A, due to the catalytic preferences of FTO to demethylation of m^6^A and m^6^A_m_ [48].

### Chemical methods to identify N^6^-methyladenosine sites

In the above methods, m^6^A sites are indirectly judged according to the adenine near the mutational point induced by RNA-antibody crosslinking or the enzyme reaction, giving rise to difficulty in the identification of real sites (especially cluster sites). Chemical methods could detect m^6^A sites in a direct way. The *N*^6^-methyl group in m^6^A has adenosine-like inert chemical property, so there are few chemical methods for the detection of m^6^A. Currently the positions of m^6^A are marked by the specific chemical reactions. *S*-adenosyl methionine (SAM) is the cofactor of methyltransferase, providing methyl donor groups for m6A biogenesis [49]. By feeding human and mouse cells with *Se*-allyl-_L_-selenohomocysteine, which transfers SAM to allyl-SAM and allyl-SeAM, cellular RNAs could be modified by *N*^6^-allyladenosine(a^6^A) instead of *N*^6^-methyladenosine at special sites [50,51].After the iodination-induced cyclization reaction [52], *N*^6^-allyladenosine could be identified as the opposite base misincorporation during reverse transcription in the high-throughput sequencing. The method called m^6^A-label-seq offers a strategy to label and identify cellular m^6^A nidification sites. However, the challenge of m^6^A-label-seq like the weak labelling and the lack of a long labelling period still leave room for it to improve. In addition, FTO-assisted m^6^A selective chemical labelling sequencing(m^6^A-SEAL-seq) [53], which combines the oxidation of m^6^A methylation by demethylase FTO with dithiothreitol(DTT)-mediated thiol-addition chemical reaction to change *N*^6^-methyladenosine to *N*^6^-dithiolsitolmethyladenosine(dm^6^A) with the free sulphhydryl group. *N*^6^-dithiolsitolmethyladenosine(dm^6^A)-marked RNA species are more stable and the free sulphhydryl group could be exploited to instal various tags like biotin and fluorophores for follow-up of the sequencing operation through reaction with methanethiosulfonate (MTSEA). These tags could be identified as m^6^A modifications during the follow-up sequencing operation. Compared to antibody-dependent MeRIP-seq, m^6^A-SEAL-seq has a similar detection resolution but more a excellent target specificity because of unbiased in the enrichment process [54]. Although chemical methods avoid antibody specificity and could identify m6A sites at higher resolution, the stoichiometric information is still unclear.

### Methods based on direct RNA sequencing to identify N^6^-methyladenosine sites

Because methylation on the RNA might be influenced by reverse transcription and PCR amplification of methods based on next-generation sequencing, direct-RNA-sequencing based on Oxford Nanopore Technologies (ONT) provided a new insight into m^6^A modification detection. ONT’ nanopore-based platform [55] uses electrophoresis to drive individual molecules through nanopores one by one for sequencing. m^6^A sites on the RNA will cause the perturbation of the current within the nanopore, making it possible to directly detect. The m^6^A sites on different isoforms of the same gene could be separated by ONT. Workman [56] proved that the median current levels at GGACU motif of m^6^A modifications which belong to different isoforms of the same gene were significantly different. Raw electric current signals are translated to RNA sequences by sophisticated base-calling software used Hidden Markov Models (HMMs) or other models [57]. Then the modified nucleoside in each position could be described. In addition, endogenous and exogenous RNA modification on long RNAs could be detected by ONT at the single-molecule level, making up the limitations on read length inherent to short-read-based methods [58]. Meanwhile, zero-mode waveguides applicated in single-molecule, real-time (SMRT) could directly obtains the information of m^6^A sites from the RNA template by following the activity of reverse transcriptase enzymes synthesizing cDNA on thousands of single RNA templates simultaneously in real time with single nucleotide turnover resolution [59]. However, the development of methods based on SMS is not yet mature and the problem of cost is not a small difficulty, but its perspective is still expected.

## Bioinformatics approached for m^6^A methylation analysis

**Figure 2.**
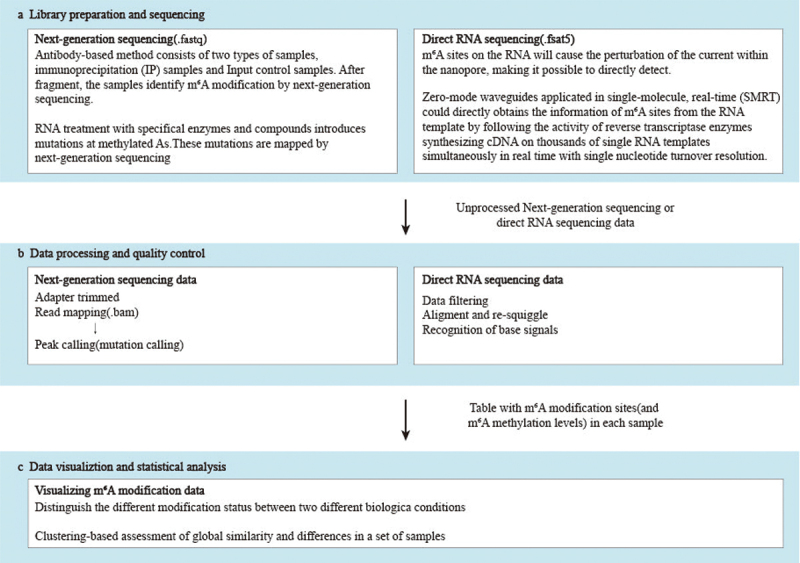
Workflow for analysing and interpreting m^6^A modification data.

The overall performance of these m^6^A Methylation Analysis methods depends not only on chemical processing and library preparation steps, but also, to a large extent, on the performance of bioinformatics pipelines and the robustness of training data sets used for parameter optimization. The methods mentioned above are mainly based on the next generation sequencing and nanopore sequencing (Figure 2). In this section, we discuss the process of bioinformatics analysis after library construction and sequencing.

### Bioinformatics analysis tools and software for MeRIP-seq(m^6^A-seq) data

#### Peak calling

As the most widely adopted experimental approach for mapping m^6^A sites on the transcriptome wide, follow-up bioinformatics analysis and data mining for MeRIP-seq are also receiving considerable attention (Table 2). The sequencing data are quality control and then aligned to the reference genome. In comparison with input samples, more read aggregations have appeared on the transcriptomic regions with m^6^A of IP samples. When visualizing the read counts along the genome, these regions are identified as peak-like shapes. The process of inferring the position of m^6^A methylation is ‘peak calling.’

**Table 2.** Bioinformatics analysis tools and software based on MeRIP-seq(m^6^A-seq) data.

Tool		Input format	URL/stand-alone package	Highlight
Peak calling	MACS2^[60]^	BAM	https://github.com/taoliu/MACS/	Genome-based ChIP-seq peak calling methods
	exomePeak2^[61]^	BAM	https://rdrr.io/github/ZhenWei10/exomePeak2/	A MATLAB package that outputs exome-based peaks (the genomic locus of RNA modification sites)
	MeTPeak^[62]^	BAM	https://github.com/compgenomics/MeTPeak	Sensitive to lowly enriched peaks
	HEPeak^[63]^	SAM	None	Perform the peak calling on connected exons of a specific gene
	m6AViewer^[64]^	BAM	http://dna2.leeds.ac.uk/m6a	Finer resolution
	MeRIP-PF^[65]^	FASTQ	http://software.big.ac.cn/MeRIP-PF.html.	Produce outputs in both XLS and graphical format
	BaySeqPeak^[66]^	Reads count matrix	https://github.com/liqiwei2000/BaySeqPeak	Outperformed when an excess of zeros are present in the data
	TRES^[67]^	Reads count matrix	https://github.com/ZhenxingGuo0015/TRES	Account for all sources of variations and alleviates the problem caused by small sample size.
	MoAIMS^[68]^	BAM	https://github.com/rreybeyb/MoAIMS	Intuitive evaluation on the treatment effect of MeRIP-seq
Differential methylation analysis	exomePeak2^[70]^	BAM	https://rdrr.io/github/ZhenWei10/exomePeak2/	Method specialized for MeRIP-seq data
	MetDiff^[71]^	BAM	https://github.com/compgenomics/MeTDiff	Significantly improved sensitivity and specificity
	MVT^[72]^	Read counts matrix	https://github.com/ouyang-lab/DIMER	Control type I error
	DRME^[73]^	Reads count matrix	https://github.com/lzcyzm/DRME	MeRIP-seq(m6A-seq) dataset in small sample cases
	QNB^[74]^	Reads count matrix	https://cran.rstudio.com/web/packages/QNB/	Improve the test performance of extremely low expression genes
	RADAR^[75]^	Reads count matrix	https://github.com/scottzijiezhang/RADAR	Consider the existence of covariates in small-sample sequencing
	bltest^[76]^	Reads count matrix	None	Need not an independent procedure to unify different library sizes of the MeRIP-Seq samples
	FET-HMM^[77]^	BAM	https://github.com/lzcyzm/RHHMM	Distinguish multiple methylated residues within a single methylated site
Clustering analysis of m^6^A sites	Binary clustering^[80]^	M-value	None	Connect m^6^A sites with different RNA functions
	MeTCluster^[81]^	BAM	http://compgenomics.utsa.edu/metcluster	Uncover the co-methylation pattern in MeRIP-seq data
	DPBBM^[82]^	Reads count matrix	https://cran.r-project.org/web/packages/DPBBM/	Adopt a nonparametric Dirichlet process to determine the optimal number of clusters
	Four clustering methods^[83]^	M-value	None	Unveil the linkage between the global RNA co-methylation patterns and the latent enzymatic regulators
	Biclustering algorithms^[84,85]^	M-value	None	Explain local co-methylation patterns (LCPs)
	REW-ISA V2^[86]^		None	Find potential local function blocks (LFBs)
	Threshold-Based Measurement Weighting^[87]^	Reads count matrix	None	Largely tolerate the various artefacts

Chromatin Immunoprecipitation following by next generation sequencing (CHIP-seq) used to analyse protein interactions with DNA has similar data composition with MeRIP-seq. Therefore, MACS2 [60] a CHIP-seq peak calling methods, has become a common peak calling method for MeRIP-seq data. Nevertheless, due to the great variation of input which measure the gene expression intensity, the methods developed for CHIP-seq data will call more peaks for highly expressed genes, generating peak calling errors. Moreover, replications in MeRIP-seq are few, which will lead to an unstable estimation of the variance. Special methods and software should be developed to analyse MeRIP-seq data. As a software tool specifically designed for MeRIP-seq data, exomePeak2 [61] is calling exome-based peaks and sensitive to PCR amplification biases universally appearing in NGS data. Based on the exomePeak2, MeTPeak [62], sensitive to lowly enriched peaks, is a graphical model-based peak calling method, improving the detection performances and robustness. Another software tool for connected exons of a specific gene is HEPeak [63] which was based on the Hidden Markov Model (HMM), improving sensitivity and detection specificity. A graphical user interface was proposed by m6aViewer [64] to reduce the difficulty of the entire process. Other approaches such as MeRIP-PF [65], BaySeqPeak [66], TRES [67] and MoAIMS [68] improved the output format and zero-inflated problems, making the presented results precise and intuitive.

#### Differential methylation analysis

Differential methylation analysis concentrates on the different modification status between two different biological conditions. There are three levels of differential analysis: differential methylation probe(DMP), differential methylation region(DMR) and differential methylation block(DMB). Because MeRIP-seq could not detect m^6^A modification at a single-nucleotide resolution, DMRs, the regions with different levels of methylation under two different conditions, are the best choice to research [69]. The comparison of differences in RNA methylome in the case-control study is the point of differential methylation analysis. Compared to DNA differential methylation analysis, the total amount of methylated RNA and the relative amount of methylated RNA might have different trends because of the uncertainty of the level of expression. Consequently, the relative abundance or methylation ratio, especially the ratio of methylation to total RNA, is the primary focus of current tools and software.

As an available computational tool specialized for MeRIP-seq, exomePeak2 [70] performed a simple Fishers precision test on normalized average read counts of two sets of IP/ control samples detected for the purest DMS region. Since MeRIP-seq read counts have significant biological and technical variations, detection based only on average read counts may introduce a large number of false positives. A β-binomial model was adopted by MeTDiff [71] to model differences in transcriptions and multiple MeRIP-seq samples, achieving a significant improvement in detection sensitivity and specificity. exomePeak2 and HEPeak are both window-based methods of estimating the methylation signal, which imposes a strong dependence structure on the reads counts across the gene. Therefore, a distribution-free test statistic was adopted by MVT [72] to control type I error, achieving high power to detect differential RNA methylation. Count-based small sample estimation of biological variability in MeRIP-seq is also a major challenge. The within-group variability was resolved with the models based on negative-binomial distribution in RNA-seq data. On the other hand, in MeRIP-seq data, within-group biological variability also has an impact on the differential RNA methylation analysis. Inspired by the similar research in RNA-seq data, the negative binomial model was used by DRME [73] for the small sample size MeRIP-seq dataset. Furthermore, 2 independent negative binomial distributions are more included in QNB [74], which could greatly improve the performance of tests for extremely low expression genes. However, the presence of covariates is not taken into consideration by both methods above. According to research needs, the covariates could be incorporated in the analysis by RADAR [75], which identify altered methylation sites by modulated the expression level before immunoprecipitation and the change of count after immunoprecipitation by different strategies. The unification of various library sizes of the MeRIP-seq samples leads to a certain loss of data information. A binomial likelihood ratio test was used by bltest [76] to identify differential methylation regions by the difference in the success rates of binomial distribution in the IP and input samples. And the prediction precision is improved, especially in the unbalanced sample library sizes. Moreover, the multiple methylated residues within a single methylation site cannot be identified effectively by existing peak-based methods. The detected m^6^A methylation site is divided by FET-HMM [77] into several adjacent small bins and models the dependence between spatially adjacent bins by the hidden Markov model. Since antibody-based approaches lack the stoichiometric information of m^6^A sites, antibody-independent methods have attracted increasing attention in differential methylation analysis. However, most antibody-independent methods pay little attention to differential methylation analysis, it still takes time to develop novel bioinformatic analysis tools based on the stoichiometric information of m^6^A sites.

#### Clustering analysis of m^6^A sites

The m^6^A sites are enriched in the transcription initiation region with high CpG genes [78], implying that m^6^A may directly regulate the epigenetic state and transcription of corresponding genes through co-transcription. Furthermore, the new phenomenon RNA epigenetic modification information flows from RNA to chromatin via co-transcription are revealed by the direct relationship between m^6^A methylation and dynamic modification of histone [79].Consequently, the associated methylation mechanisms between the m^6^A function could be explained by the clustering analysis of m^6^A sites. The m^6^A methylation sites are classified by Schwartz [80] as WTAP-dependent and independent sites that connected to different RNA functions by binary clustering, respectively. WTAP-dependent sites are located at internal positions in transcripts, and its topology is relatively static across mRNAs. The co-methylation pattern in MeRIP-seq data is discovered by MeTCluster [81] with the cluster for the degree of m^6^A methylation peaks based on the expectation-maximization algorithm, providing a new direction for studying the mechanisms and functions of m^6^A modification. However, the low reads coverage of count-based RNA methylation sequencing data might influence the cluster result. Therefore, a beta-binomial mixture model [82] was proposed to capture the clustering effect in methylation level and a nonparametric Dirichlet process was adopted to determine the optimal number of clusters. The method not only reveal novel m^6^A co-methylation patterns from sites of very low reads coverage, but also learns an optimal number of clusters adaptively from the data analysed. On the other hand, the linkage between the global RNA co-methylation patterns and the latent enzymatic regulators are unveiled by the clustering approaches [83] including K-means, Hierarchical clustering (HC), Bayesian factor regression model (BFRM) and nonnegative matrix factorization (NMF), providing a promising prospect for researching of the epitranscriptome. However, the detailed regulatory mechanisms for m^6^A methylation sites under different conditions remain unclear. The phenomenon which the methylation levels of certain sites increase or decrease simultaneously under certain conditions and sites from the same co-methylation module exhibit simultaneous hypermethylation or hypomethylation under certain conditions could be explained by the local co-methylation patterns (LCPs). The phenomenon which the methylation levels of certain sites increase or decrease simultaneously under certain conditions and sites from the same co-methylation module exhibit simultaneous hypermethylation or hypomethylation under certain conditions could be explained by the local co-methylation patterns (LCPs). The situation could be regarded as a typical biclustering problem, which is widely used in the discovery of co-expression patterns. Biclustering algorithms, FBCwPlaid [84] and BDBB [85], have been proposed to mine LCPs of m6A epi-transcriptome data. Furthermore, an RNA Expression Weighted Iterative Signature Algorithm are adopted by RNREW-ISA V2 [86], revealing the potential local functional patterns presented in m6A profiles, where sites are co-methylated under specific conditions. Compared with ordinary biclustering algorithms, overlapping local functional blocks (LFBs) in the input data could be obtained by REW-ISA V2. Moreover, the various high-throughput sequencing data artefacts induced by the intrinsic properties of RNA molecules, RNA modification and other influence elements could be tolerated based on a measurement weighting strategy [87].

### Bioinformatics workflow based on reverse transcription (RT) signatures

Reverse transcription (RT) signatures are caused by modifications on the Watson-Crick face [88]. As mentioned in the above experimental methods, combined with the high-throughput sequencing, RT signatures occurred during the synthesis of complementary DNA (cDNA) is an effective mark to the analysis of m^6^A methylation. There are several library preparation protocols for capturing cDNA with RT signatures [89–91]. HAMR [92] was regarded as one of the first attempts to adopt the RT signatures to analysis the modifications in the RNA template. However, there is a lack of specialized visual function in early-stage viewers. The CoverageAnalyzer(CAn) did an automated screening of candidates for modification and a detailed visual inspection of RT signatures [93].And the typical CAn session provides data sorting and filtering options, visualization tab, candidate casting tab, formula editor and control panel for the serial trace. In addition, a versatile graphical workflow system for modification calling based on machine learning models was presented by Galaxy [94], which are equipped with the principal module (trimming, mapping and postprocessing) and other downstream modules. It should be noted that the detection of modifications by RT signatures can only be applied to a limited subset of given RNA modifications, as many modifications do not affect the Watson-Crick base pairing during cDNA synthesis, which called RT silent [95]. Moreover, the biases and artefacts because of the conversion from RNA to cDNA will be introduced by the mapping approaches using RT signatures of mRNA and polymerase chain reaction (PCR) [96]. These problems could be resolved by the direct long reads sequencing of Oxford Nanopore Technologies (ONT).

### Bioinformatics analysis of nanopore sequencing

The perspective of mapping m^6^A methylation approaches by nanopore technology is luciferous. However, the raw signals counterpart to the sequence contexts of modified and unmodified base are still inexplainable until now. As a result, currently available algorithms that map RNA modification by nanopore sequencing are based on either modification-induced base calling errors or difference in raw signal levels (Table 3).

**Table 3.** Bioinformatics tool for nanopore sequencing.

Tool		Feature	Algorithm	URL/stand-alone package
Deviation of current signal between WT and WD	ELIGOS^[97]^	The percent Error of Specific Bases(%ESB)	None	https://gitlab.com/piroonj/eligos2
	EpiNano^[98]^	Differential base-calling errors	Nanopolish	https://github.com/enovoa/EpiNano
	DiffErr^[99]^		None	https://github.com/bartongroup/differr_nanopore_DRS
	DRUMMER^[100]^	Matches/Mismatches rates	None	https://github.com/DepledgeLab/DRUMMER
Differences in raw current intensity	MINES^[101]^	Methylation values from Tombo denovo	Tombo	https://github.com/YeoLab/MINES
	Nanocompore^[103]^	Signal intensity, dwell time	Nanopolish	https://github.com/tleonardi/nanocompore
	xPore^[104]^	Signal intensity	Nanopolish	https://github.com/GoekeLab/xpore
	Yanocomp^[105]^	Signal intensity	Nanopolish	https://github.com/bartongroup/yanocomp

The first category of algorithms concentrates on the error frequencies as compared to the background at specific sites in the transcriptome. The epitranscriptional landscape from glitches of ONT signals the percent Error of Specific Bases(%ESB) of native RNA has been found higher than unmodified RNA. ELIGOS [97] infers the epitranscriptional landscape from glitches of ONT singnals, which is appropriate for various types of synthetic modified RNA. However, this method is influenced by the different nanopore motor, sequencing directions, and basecalling models between RNA and cDNA. Furthermore, based on differential base-calling errors, EpiNano [98] and DiffErr [99] were developed to identify m^6^A sites in paired conditions (wild type and METTL3 KD). Meanwhile, the ribonucleic acid modifications also could be detected is by DRUMMER [100] used the frequency of matches/mismatches between a WT and a low modified sample. Nevertheless, due to the single error is not available to confirm the presence of modification, the methods based on modification-induced base calling errors provide the location of multiple modified sites, but could not achieve the detection at single-molecule resolution.

The second category of approaches is based on the raw current/signal intensity. The most common m^6^A sequence context, DRACH, was focused by MINES [101]. The Tombo’s de novo detection algorithm [102] used by MINES provides coverage of genomic reference yield only along the 3’ untranslated regions (UTRs). It could be supplied in combination with the cDNA reference. The modification stoichiometry of 30 nucleotide windows centred on the A of each DRACH motif with a minimum read coverage of 5 reads is filtered. And the Random Forest Model (RFM) is used to classify the central A of the DRACH motif as modified or unmodified by these stoichiometry values. Moreover, the comparation between the raw electrical signal for a sample of interest and the signal in a control sample containing fewer or no modifications are utilized by Nanocompore [103] to detect potential RNA modifications. The significant difference in the distribution of reads into the two clusters between conditions is distinguished by a bivariate classification method based on two components Gaussian mixture model (GMM) clustering followed by a logistic regression test (logit). In addition, a multi-sample two Gaussian mixture model with the prior of the theoretical signal distribution of the unmodified k-mer is adopted by xPore [104]. It is considered a significant innovation to attribute two distributions obtained from GMM to be assigned to a modified or unmodified state. The essence of xPore is to model the probability for each modified read, which could be used to calculate the fraction of reads that are assigned to the modified signal distribution. To improve accuracy and reduce false positives, similar positions where distributions for unmodified and modified signals and the one-directional signal shifts RNA modification induced the signal for each k-mer must be considered. Furthermore, the multivariate Gaussian mixture model is used by Yanocomp [105] to model the average ionic current amplitude of the five k-mers sliding window. Outliers are represented by two Gaussian components and a third uniform component to minimize overfitting.

Similar to m^6^A modification, ONT is also applicable for the detection of other RNA modification like A-to-I [106,107], Ψ [108] and m^5^C. However, the detection might be effect by the species of RNA modification and other factors.

## Computational Methods for N6-Methyladenosine Sites Prediction

**Figure 3.**
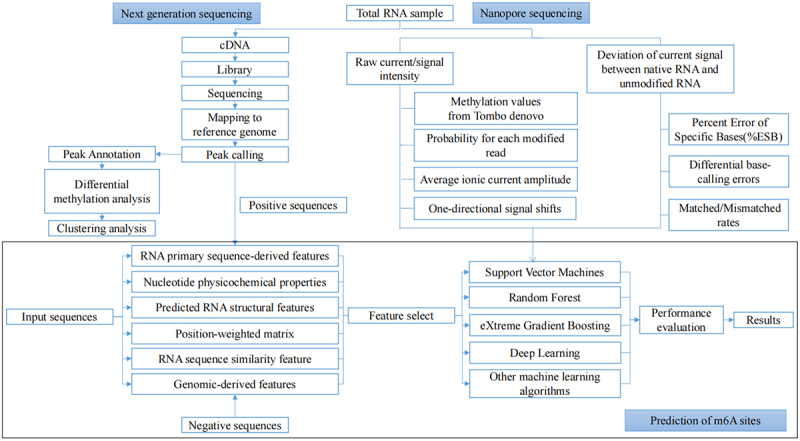
The overview of bioinformatics methods to decipher the m6A methylation.

Given that experimental approaches are expensive and limited, *in silico* methods have attracted increasing attention as an alternative means of studying m^6^A modification. (Table 4) The data produced by experimental methods provide benchmark datasets for model construction. Most m^6^A sites prediction methods and web servers extracted input features from the sequence-derived information and other genomic information and predicted m^6^A sites by various machine learning approaches. Finally, the performance of the measures is evaluated (Figure 3).

**Table 4.** Summary of the reviewed predictors for m^6^A sites.

Tool	Dataset modes	Feature encoding	Feature selection	Algorithm	Evaluation strategy	Web server
WHISTLE^[119]^	Both	Nucleotide chemical property Cumulative nucleotide frequency Genome-derived features	Perturb method	SVM	Fivefold cross-validation Independent test	https://whistle-epitranscriptome.com
iRNA-m6A^[120]^	One	Physical-chemical property matrix Mono-nucleotide binary Nucleotide chemical property	mRMR	SVM	5-fold cross-validation	https://lin-group.cn/server/iRNA-m6A
MethyRNA^[121]^	Both	Nucleotide chemical property Accumulated nucleotide frequenc	None	SVM	Jackknife test	https://lin.uestc.edu.cn/server/methyrna
SRAMP^[122]^	Both	Positional binary KNN score Nucleotide pair spectrum	None	RF	Fivefold cross-validation	https://www.cuilab.cn/sramp/
HMpre^[123]^	One	Positional binary	None	XGBoost	10-fold cross-validation	None
M6AMRFS^[124]^	One	Dinucleotide Binary Local Position-Specific Dinucleotide	SFS	XGBoost	10-fold cross-validation jackknife test	https://server.malab.cn/M6AMRFS/
Gene2vec^[125]^	One	One-hot Neighbouring methylation state RNA word embedding	None	CNN	Training and validation	https://server.malab.cn/Gene2vec/
BERMP^[126]^	One	Enhanced Nucleic Acid Composition RNA word embedding	None	BGRU	10-fold cross-validation	https://www.bioinfogo.org/bermp
EMDLP^[132]^	One	RNA word embedding, One-hot encoding, RGloVe	None	NLP DL	Independent validation	https://www.labiip. net/ EMDLP/ index. php (https://47.104.130.81/EMDLP/ index. php)
EDLm^6^APred^[131]^	One	One-hot RNA word embedding Word2vec^[176]^	None	NLP DL	Independent validation	https://www.xjtlu.edu.cn/biological sciences/ EDLm6 APred
WeakRM^[134]^	One	One-hot	None	DL	validation approach based on low-resolution data	https://github.com/daiyun02211/WeakRM
m6A-Maize^[135]^	One	One-hot	None	DL	fold cross-validation	https://www.xjtlu.edu.cn/biologicalscien ces/maize.
MultiRM^[136]^	One	One-hot Seq2vec^[177]^ Word2vec	None	CNN RNN	Independent validation	https://www.xjtlu.edu.cn/biologicalsciences/multirm
DeepM6ASeq^[137]^	One	One-hot	None	DL	Independent validation	https://github.com/rreybeyb/DeepM6ASeq

### Dataset construction and feature extraction

The positive sample (m^6^A sites) and the negative sample are extracted according the published maps of m6A sites. And the potential influence of sequence redundancy could be removed by the CD-HIT-EST tool [109]. The redundant sequence is defined as the training sample with high sequence identity compared to another testing sample. The threshold of sequence similarity ranged commonly from 60% to 80%. In addition, the bench-mark datasets are built by two modes. The genomic sequences are used by the full transcript mode as an input and the cDNA sequences are considered by the mature mRNA mode instead. However, most methods consider only one of these patterns.

In order to construct robust and precise machine learning predictors for predicting RNA modification sites, multiple features were designed and extracted to encode RNA sequences. In the computational approaches published at present, features are mainly divided into six categories [110], including RNA primary sequence-derived features, nucleotide physicochemical properties, predicted RNA structural features, position-weighted matrix, RNA sequence similarity feature and genomic-derived features. Among them, the primary sequences with the most fundamental biomolecules information are most versatile features for developing approaches for RNA modification. Numerous tools have been developed for feature extraction and modelling of primary sequences, such as BioSeq-Analysis [111,112], PyFeat [113] and BioSeq-BLM [114]. However, high dimensional vectors may result in large computation, overfitting and low robustness of the proposed model. Therefore, the selection of feature is indispensable to exclude noise and improve the computational efficiency of the models. The mRMR algorithm [115] is usually used to acquire the optimal feature subset because of its usability and efficiency. Furthermore, the perturb method and the SFS are also used for feature selection.

### m^6^A methylation site prediction

Most of the RNA modification prediction methods are based on machine learning models. To date, almost 100 different approaches [116–118] have been established, including notably, WHISTLE [119], iRNA-m6A [120], MethyRNA [121], SMAMP [122], HMpre [123], M6AMRFS [124], Gene2vec [125], BERMP [126],etc. Among them, WHISTLE predicts m^6^A sites based on the deep learning models. Because both the transcript structure and the relative position on the transcript are found to be related to the occurrence and function of RNA sub-molecular events, transcript annotation could be used as an information source for predicting m^6^A modification [127,128]. Therefore, geographic encoding of transcripts might be used for deep learning models applied to RNA transcripts. Compared to other deep learning models, the transcript region information incorporated into genomic features by WHISTLE greatly improves its performance. However, information regarding the position relative to the boundaries of the long-range is neglected. In addition, one-hot encoding is widely used to describe the transcript region [129], but it may result in an incomplete landscape of the local transcript structure. To fill the gap, three novel encoding methods, landmarkTX, gridTX, and chunkTX, were developed by Geo2vec [130]. Combined with one-hot encoding, more informative and interpretable sub-molecular geographic descriptors of transcripts are provided. Furthermore, experimental results indicate that the base, upstream, and downstream information of m^6^A sites are all critical to detection. Natural language processing is used to feature extraction and classification of m^6^A methylation sites with consideration of context information [131–133] Most of above methods are based on strong supervision enabled by base-resolution data, but in some situation low-resolution m^6^A modification data can only be obtained. Weakly supervised learning models [134,135], which learns from datasets produced by low-resolution experimental methods like MeRIP-seq, were proposed to predict the situation of m^6^A modification. Moreover, the precise identification of all types of RNA modification is essential for understanding the functions and regulatory mechanisms of RNAs. The relationship among different types of RNA modifications was revealed by MultiRM [136], allowing an integrated analysis of common RNA modifications. In addition to predicting m^6^A-containing sequences, the biological features surrounding m6A could be characterized to elucidate its regulatory code [133,137]. Besides, conservation analysis of individual m6A sites is achieved by a novel scoring framework, ConsRM [137]. ConsRM has been confirmed to outperform phastCons [138], a traditional and versatile conservation score, in distinguishing conserved m^6^A sites.

### Performance evaluation measures and strategies

Sensitivity (Sn), Specificity (Sp), Accuracy (Ac) and Matthews Correlation Coefficient (MCC) are used to measure the predictor’s performance at certain thresholds. They are defined as:

(1) $$Ac=\frac{TP+TN}{TP+FN+TN+TP}$$

(2) $$Sn=\frac{TP}{TP+FN}$$

(3) $$Sp=\frac{TN}{TN+FP}$$

(4) $$MCC=\frac{TP\times TN-FP\times FN}{\sqrt{\left(TP+FP\right)\times \left(FP+FN\right)\times \left(TN+FN\right)\times \left(TN+FP\right)}}$$

Where TP, FP, TN and FN each represent the true positive, false positive, false negative and true negative predictions, respectively. Receiver-operating-characteristic (ROC) curves for the predictors and the area under ROC curve (AUC) are also calculated to evaluate the overall performance of the predictors. The higher the AUC and AUPRC value, the better the prediction performance.

Three validation methods, including the K-fold cross-validation test, jackknife validation test and independent dataset test, are often used to derive comparative metrics (values) among the reviewed predictors. Generally, the jackknife is commonly used in the prediction task with a smaller dataset size, while K-fold cross-validation and independent tests are most popular in the prediction task with a larger dataset size. Of the reviewed predictors, most carried out 1–2 cross-validation tests.

## Identification of m^6^A modification in circRNAs

Circular RNA (circRNA) was first discovered in pathogens in 1976 [139] and soon afterwards in eukaryotic cells [140]. It is a class of single-stranded covalently closed RNA molecules generated by back-splicing [141]. A growing body of research has confirmed that circRNAs play a critical role in the occurrence and development of various diseases [142–144]. The m^6^A modification is the most common and well-studied post-transcriptional RNA modification pattern across almost every type of RNA molecules including circRNA [145]. Currently, the research on m^6^A modification in circRNA is in an incipient state. Growing evidence has corroborated that m^6^A modification is critical to regulate the metabolism and functions of circRNAs [146,147]. The approaches mentioned in section 2 could also be used to detect m^6^A modification in circRNAs. For example, MeRIP is still the principal method for analysing the m^6^A level of circRNA [148,149], and circRNA-m^6^A-seq could identify the m^6^A-containing endogenous circRNAs after treating the RNA sample with exoribonuclease RNase R [150]. Meanwhile, computational methods are developed based on the MeRIP-seq data to map m^6^A modifications in circRNA [151,152]. In addition, due to its unique property of low total RNA input, m^6^A-circRNA epitranscriptomic microarray [153,154] is coming into vogue. However, microarray based on the hybridization of probes must have the sequence of the samples in advance. Moreover, ‘silicon-on-insulator’ structures were used to fabricate biosensor-based chips [155,156] to detect the m^6^A modification in circRNA. Despite this method holds characteristics of sensitivity, stability and real-time, the reliability of identification is low. The relationship between circRNAs and m^6^A is not clear at this point, the detection of m^6^A modification in circRNAs remains difficult.

## m^6^A methylation databases

Knowledgebase with the comprehensive collection and integration of various information related to m^6^A modification is of crucial importance for elucidating the functional and regulatory circuitry of it as well as for developing bioinformatics tools(Table 5). To date, more than a million m^6^A methylation sites have been found from studies between different species. These sites and other types of modification sites are all contained in RMBase [157,158]. The online visualization tool is also developed by RMBase to plot modification motifs and metagenes of RNA modification along a transcript model. As another database with the location of modified residues, MODOMICS [159,160] is currently the most comprehensive source of RNA modification pathway. In the latest version, the new external resources like RCBS Protein Data Bank database(links to structure), Human Metabolome Database [161], PubChem [162] and ChEMBL [163]. Compared to RMBase, only 442,162 m^6^A sites are identified by m^6^A-Atlas [164], but these sites with high-confidence are based on different base-resolution technologies and the quantitative epitranscriptome profiles estimated from high-throughput sequencing samples. Moreover, the biological functions of individual m^6^A and the potential pathogenesis of m^6^A could be predicted by m^6^A-Atlas from epitranscriptome data. On account of its function as a diseases (including various cancers) regulatory factor, the m^6^A modification is considered as a developing guideline for the treatment of targeted disease. 222 experimentally confirmed m^6^A-disease association are included by M^6^ADDsites and the potential pathogenesis of m^6^A could be predicted by m^6^A-Atlas from epitranscriptome data. On account of its function as a diseases (including various cancers) regulatory factor, the m^6^A modification is considered as a developing guideline for the treatment of targeted disease. 222 experimentally confirmed m^6^A-disease association are included by M^6^ADD [165] to explore the associations between m^6^A modification and gene disorders and diseases. Furthermore, it remains a major challenge to distinguish disease-causing variants among a large number of single nucleotide varoants(SNP). The m^6^A-associated variants that potentially influence m^6^A modification are integrated by m^6^Avar [166], deriving from three different m^6^A sources including miCLIP/PA-m^6^A-seq experiments (high confidence), MeRIP-seq experiments (medium confidence) and transcriptome-wide predictions (low confidence), to evaluate the effect of variants on m^6^A modification. The data on the effects of m^6^A-centred regulation on both disease development and drug response are covered by M^6^AREG [167]. As is mentioned above, a variety of experimental methods have been developed for m^6^A site detection. Among them, MeRIP-seq is the earliest as well as the most popular method. REPIC [168] and MeT-DB [169] are specifically for recording peaks called from MeRIP-seq data. In addition, all of the above databases focus on the relationships between m^6^A modification and RNA-binding proteins (RBPs). A series of regulatory factors, m^6^A writers, erasers and readers (WERs), play a role in the dynamic and reversible process of m^6^A modification. Due to different downstream genes targeted by the WERs, the same WER might perform different functions under different conditions. The known m^6^A WER target genes are hosted by M^6^A2Target [170] to elucidating the function of m^6^A dynamic modification.

**Table 5.** m^6^A methylation databases.

Database	Main content	Availability
RMBase^[157,158]^	The most comprehensive collection of RNA modification sites	https://rna.sysu.edu.cn/rmbase/
MODOMICS^[159,160]^	The most comprehensive source of RNA modification pathway	https://iimcb.genesilico.pl/modomics/
m^6^A-Atlas^[164]^	m^6^A sites with high-confidence	https://www.xjtlu.edu.cn/biologicalsciences/atlas
M6ADD^[165]^	Experimentally confirmed m6A-disease association	https://m6add.edbc.org/
M6AREG^[167]^	The effects of m^6^A-centred regulation on both disease development and drug response	https://idrblab.org/m6areg/
REPIC^[168]^	Peaks called from MeRIP-seq data	https://epicmod.uchicago.edu/repic/index.php
MeTDB^[169]^	Peaks called from MeRIP-seq data	http://rna.sysu.edu.cn/rmbase/
M6A2Target^[170]^	The known m^6^A WER target genes	http://m6a2target.canceromics.org

## Validation method for *N*^6^-Methyladenosine sites

Different high throughput techniques are prone to different degrees of error. Current datasets including hundreds to thousands of sites, were primarily predicted using high-throughput sequencing data(Table 6). With the increasing of published datasets, a great number of detected m^6^A sites might be highly uncertainty. It’s necessary to regard detected m^6^A sites as candidate sites and validate the datasets based on high-throughput sequencing methods by at least one additional independent method. The greater the difference between the nature of the validation method and the original method, the higher the reliability of the validation [88]. As an early way to identify m^6^A modifications, liquid chromatography-tandem mass spectrometry (LC-MS/MS) [171] analysis the m^6^A methylation via neutral loss Scan (NLS) and dynamic multiple reaction monitoring(DMRM).The enriched RNA also could be directly quantified by quantitative PCR used by Methyl-RNA-immunoprecipitation-quantitative PCR(MeRIP-qPCR) after m^6^A antibody is enriched to RNA with methylation modification [172]. But exact measurement of the m6A stoichiometry at the specific site is irrealizable in LC-MS/MS and MeRIP-qPCR. To solve the situation, site-specific cleavage and radioactive-labelling followed by ligation-assisted and thin-layer chromatography (SCARLET) [173] could accurately determine m^6^A status at any site in mRNA/LncRNA. Moreover, the significant selectivity against m^6^A modification of T3 DNA ligase has been found [174], which could be used to determine the m6A modification fraction at the precise location. In addition, SELECT [175] exploit the decrease of the single-base elongation activity of DNA polymerases and the nick ligation efficiency of ligases hindered by m^6^A, quantifying by qPCR.

**Table 6.** Validation Method for *N*^6^-Methyladenosine Sites.

Methods	Advantages	Limitations
LS-MS/MS^[171]^	Standardized method Easy preparation steps	Inability to distinguish m^6^A on mRNA from m^6^A on rRNA or snRNA contaminants
MeRIP-qPCR^[172]^	Semi- stoichiometric information at specific m^6^A sites Low amount of input material required	Inability to distinguish stoichiometry of adjacent m^6^A sites Need for external methylated and non-methylated spike- in controls
SCARLET^[173]^	Exact measurement of the m^6^A stoichiometry at the specific site	Low throughput Ability to detect only one site per transcript at a time
T3/T4DNA ligase-qPCR^[174]^	Easy preparation steps Potential measurement of m6A stoichiometry	Dependence on efficiency of the ligation reaction Low throughput
SELECT [175]	Easy preparation steps Measurement of m6A stoichiometry	Dependence on two selective steps: efficiency of the Bst polymerase and efficiency of the ligation reaction

## Conclusion and discussion

With increasing emphasis on Epitranscriptome, the bioinformatics capacity to analyse, digest, collect and share the rapidly growing epitranscriptome profiling data is sorely needed. The review summarizes the recent topics and progress made in bioinformatics methods of deciphering the m^6^A methylation, including the experimental detection of m^6^A methylation sites, techniques of data analysis, the way of predicting m^6^A methylation sites, m^6^A methylation databases, and detection of m^6^A modification in circRNA. Together, developments in bioinformatics have greatly facilitated research in the field and have improved understanding of the biological significance of RNA modifications.

Nevertheless, in spite of the rapid advances in epitranscriptome bioinformatics, there are still a number of limitations or open questions. The technological bias and limitations may not have received sufficient attention when developing bioinformatic tools. The issues of cost and efficiency remain the limitations of many methods, particularly the Oxford nanopore method. Furthermore, some bioinformatics pipelines have not been expended to accommodate the emergence of new modifications resulting from the development of new technologies. In addition, the crosstalk between circRNAs and m^6^A is in the incipient stage and remains challenging. And the user-friendly, freely accessible and comprehensive database integrating m^6^A-circRNA-related research is still not developed for the studies the relation of m^6^A and circRNA. Lastly, with the rapid development of single-cell DNA methylation, single-cell m^6^A sequencing is beginning to be a novel breakthrough in the study of m^6^A landscape and function.
